# Radial artery harvesting in coronary artery bypass grafting surgery—Endoscopic or open method? A meta-analysis

**DOI:** 10.1371/journal.pone.0236499

**Published:** 2020-07-24

**Authors:** Tzu-Yen Huang, Ting-Shuo Huang, Yu-Ting Cheng, Yao-Chang Wang, Tzu-Ping Chen, Shun-Ying Yin, Chi-Hsiao Yeh

**Affiliations:** 1 Department of Thoracic and Cardiovascular Surgery Chang Gung Memorial Hospital at Keelung, Chiayi, Taiwan; 2 Department of Biomedical Engineering, National Taiwan University, Taipei, Taiwan; 3 Department of General Surgery, Keelung Chang Gung Memorial Hospital, Taoyuan City, Taiwan; 4 Community Medicine Research Center, Keelung Chang Gung Memorial Hospital, Taoyuan City, Taiwan; 5 Department of Chinese Medicine, College of Medicine, Chang Gung University, Taoyuan City, Taiwan; 6 Department of Thoracic and Cardiovascular Surgery, Chang Gung Memorial Hospital at Linkou, Taoyuan City, Taiwan; 7 College of Medicine, Chang Gung University, Taoyuan City, Taiwan; Hoffman Heart Institute of the Saint Francis Hospital and Medical Center, UNITED STATES

## Abstract

We analyzed the clinical outcomes of open radial artery harvesting (OAH) and endoscopic radial artery harvesting (EAH) undergoing coronary artery bypass grafting (CABG). We designed this meta-analysis conducted using Pubmed, Medline, the Cochrane Library, and EMBASE. Articles with comparisons of OAH and EAH undergoing CABG were included. Primary outcomes included the wound infection rate, the wound complication rate, neurological complications of the forearm, in-hospital mortality, long-term survival, and the patency rate. The results of our study included six randomized controlled trials (RCTs), two non-randomized controlled trials (NRCTs) with matching, and 10 NRCTs. In total, 2919 patients were included in 18 studies, while 1187 (40.7%) and 1732 (59.3%) patients received EAH and OAH, respectively. EAH was associated with a lower incidence of wound infection (RR = 0.29, 95% confidence interval (CI) = 0.14 to 0.60, *p* = 0.03), and neurological complications over the harvesting site (RR = 0.41, 95% CI = 0.27 to 0.62, *p* < 0.0001). There was no significant difference in 30-day mortality, long-term survival (over one year), and the graft patency rate. According to our analysis, endoscopic radial artery harvesting can improve the outcome of the harvesting site, without affecting the mortality, long-term survival, and graft patency.

## Introduction

Coronary artery disease is one of the most common cardiac diseases, with an in-hospital mortality rate of 6%–8% [1]. Previous study has shown that in patients with ST-elevation myocardial infarction (STEMI), in-hospital mortality rates for patients receiving coronary artery bypass grafting (CABG; 3.52%) or percutaneous coronary intervention (PCI; 5.70%) were significantly lower than those without intervention (14.91%, *p* < 0.001) [2]. In patients with non-ST-elevation myocardial infarction (NSTEMI), the in-hospital mortality rates for patients receiving CABG (1.45%) or PCI (2.91%) were also significantly lower than those without intervention (6.26%, *p* < 0.001) [2]. Revascularization with either CABG or PCI is essential for decreasing the mortality of patients with STEMI or NSTEMI.

CABG surgery is still the golden standard of treatment for severe and complex coronary artery diseases [3]. Despite recent progress in the technology of PCI, CABG still has a higher patency rate and lower mortality rate after 1-year and 5-year follow-up [4, 5]. Furthermore, it was indicated by a study with 7-year follow-up that multiple artery graft CABG had a significantly lower mortality rate (12.7% vs. 14.3%, *p* < 0.001), less repeat revascularization (11.7% vs. 14.6%, *p* < 0.001), and fewer major adverse cardiac events (20.2% vs. 22.8%, *p* < 0.001), compared with single artery graft CABG, especially for higher-volume surgeons [6]. The bilateral internal thoracic artery, radial artery (RA), right gastroepiploic artery, and ulnar artery can be used as the arterial conduit for CABG, while the radial artery is one of the most common arterial conduits [7].

The harvesting of RA as a CABG conduit was first introduced in the 1970s by Carpentier et al. [8]. However, it was abandoned after 1976 due to its low patency rate [9, 10]. It was reintroduced and its use was revived by Acar et al. in 1992 [11]. Previous research had reviewed and discussed different methods of free vessel grafts (radial artery or great saphenous vein) harvesting before 2010[12, 13]. Nowadays, radial artery harvesting for CABG has become routine in several hospitals around the world. The open none-touch method of RA harvesting (OAH), modified by Reyes et al. [14], has been well-adopted among cardiac surgeons. Endoscopic RA harvesting (EAH) has also been developed in recent years [15]. In recent research comparing the patency rate of radial artery and great saphenous vein (GSV) grafts, RA grafts significantly lowered the risk of occlusion at long-term follow-up (50 ± 30 months, hazard ratio, 0.44; 95% confidence interval (CI), 0.28 to 0.70; *p* < 0.001), especially for patients under the age of 75 [16]. However, whether the approach and quality of radial artery harvesting play an important role in graft patency deserves further clarification. Our review will discuss the clinical outcomes of OAH and EAH.

## Materials and method

### Search strategy

This systematic review was registered at PROSPERO (ID: CRD42020146718), with PROSPERO published protocol and analysis planning (http://www.crd.york.ac.uk/Prospero/). The protocol of our study was also published on protocol.io (dx.doi.org/10.17504/protocols.io.bhrdj526). Searches were not restricted by the study design, language, or publication status. Studies before 1973 was excluded. The search keywords included endoscopic, tunnel, coronary artery bypass surgery, and CABG. In addition, MeSH terms were explored. The final results were combined with the following keywords: radial artery and harvest. The databases we used to conduct our searches were Pubmed, Medline, the Cochrane Library, and EMBASE, which we employed to find studies published from January 1974 to July 2019. The comprehensive search strategy is provided in S1 Table. Databases of clinical trials, conference abstracts, and reference lists of reviews were also searched to identify additional eligible trials. Our review was conducted according to the Preferred Reporting Items for Systematic Reviews and Meta-Analysis (PRISMA) statement [17]. The PRISMA checklist of our study is provided in S2 Table.

### Study selection

In the literature search, titles and abstracts of studies identified were independently screened by four authors (T.-Y.H., T.-S.H., Y.-T.C., and C.-H.Y.). After searching, these four authors finished primary selection and discussed which articles should be included in our study. Articles with comparisons of OAH and EAH were included as long as there were adult patients with coronary artery disease undergoing CABG, regardless of whether this was on-pump or off-pump. Systemic-reviews, case series, case reports, and trials without comparisons of OAH and EAH were excluded. Studies comparing OAH and minimal invasive radial artery harvesting were also excluded. Randomized controlled trials (RCTs) and non-randomized observational articles including prospective or retrospective studies were included. Primary outcomes included the wound infection rate, the wound complication rate, harvesting site neurological complications of the forearm during hospitalization, in-hospital mortality, long-term survival (over one year), and the patency rate (duration may be defined differently by research, from in-hospital to 3–5 years). Neurological complications over the harvesting site was defined as the impairment of sensitivity and mobility of the harvest sites. We reviewed the methodologies, data volume, data source, and results, as well as the author backgrounds, to identify duplicate patient groups. On the occasion of studies using duplicate patient groups, we only kept one set of patient groups as our samples for statistical analysis.

### Data extraction

Characteristics of studies (year of publication, study period, study design and setting, method of recruitment, inclusion and exclusion criteria in each study, and outcome measurement), participants (age, gender, heart function, and underlying disease), harvesting technique (harvesting time, wound size, length of radial artery, operative techniques and devices, and air infusion assisting), comparisons (types of control group), and outcomes were recorded. Multiple arm designs were used in our study, and head-to-head comparison data were extracted for data synthesis. Long-term survival (over one year) and the patency rate were evaluated by the odds ratio and variance.

### Assessment of risk of bias

Data extraction and article quality assessment were performed by three authors (T.-Y.H., Y.-T.C., and C.-H.Y.) independently. A fourth author (T.-S.H.) was consulted for disagreement settlement and quality assurance. The risk of bias of randomized controlled trials was evaluated by the Cochrane Collaboration risk-of-bias tool—Revised Cochrane risk-of-bias tool for randomized trials (RoB 2), 2018-beta-v6-25-June-2019, which is utilized for the determination of methodological quality [18]. The methodological quality of non-randomized controlled trials (NRCTs) was assessed by the Newcastle–Ottawa Quality Assessment Scale (NOS) [19]. Studies assessed with NOS ≧ 7 points were defined as high-quality studies, whereas other studies were considered low-quality studies.

### Data synthesis and statistical analysis

For the outcomes of the wound infection rate, the wound complication rate, neurological complications of the harvesting site, and in-hospital mortality or 30 day mortality were analyzed as dichotomous data. For analyzing outcomes of long-term survival (over one year) and the patency rate, hazard ratios (HRs) with 95% CIs were extracted in primary studies with survival analysis. We analyzed and measured the effects by the risk ratio (RR), with a random effects analysis model. The overall effect of treatment was estimated by the data size. If *p*-values and hazard ratios were not provided, we calculated according to the original data. The *p*-value was calculated using the Chi-square test with Microsoft Office Excel 2016. The effect size was calculated in accordance with the methods suggested by Parmar et al. [20], by using a spreadsheet developed by Tierney et al. [21]. Clinical heterogeneity was assessed by comparing the methodologies and protocol of the included studies. Statistical heterogeneity was assessed with the Chi-square test results (using a cutoff value of *p* < 0.10), and the I^2^ statistic, where I^2^ < 25%, 25% ≦ I^2^ ≦ 50%, and I^2^ > 50% indicate mild, moderate, and substantial heterogeneity, respectively [22, 23]. Subgroup analysis based on study types was divided into three groups: RCT, NRCT with propensity score matching, and NRCT. Data synthesis and statistical analysis were conducted using Review Manager (RevMan Version 5.2.6; The Nordic Cochrane Center, Copenhagen, Denmark). A funnel plot was created to evaluate publication bias, and the significance level was set at 0.05.

## Results

### Search result

#### Search result and study characteristics

Overall, 9 RCTs [24–32], 3 NRCTs with propensity score matching [33–35], and 12 NRCTs [36–47] were included. All studies included were published in English. Inevitably, we identified a total of ten articles using duplicate patient groups respectively. Among the ten articles, four articles—Kiaii et al. (2017), Burns et al. (2014), (2015), and Kiaii et al. (2013) [25–28]—shared the same patient groups, but showed different aspects of the data. We counted them as one single set of patient groups to be included as our samples (Kiaii et al. (2017)/Burns et al. (2015)). We adopted the same approach for the remaining six articles, of which two (Navia et al. (2012) and Navia et al. (2011)) [34, 35], two (Bleiziffer et al. (2008) and Bleiziffer et al. (2007)) [42, 44], and two (Ito et al. (2011) & (2011), the same authors had published two articles with the same data in 2011)) [36, 37] articles, respectively, shared duplicate patient groups. Only one article among those sharing the same data was kept for further analysis. Therefore, in total, six articles were excluded.

Shapira et al. published two studies in 2006, of which one was an RCT (Shapira 2006a) [32] and the other was a retrospective study (Shapira 2006b) [45]. Grus et al. (2011) divided all patients into three groups by the radial artery harvesting method: endoscopic harvesting, open harvesting, and mini-invasive harvesting [30]. The patients in the mini-invasive harvesting group were excluded from our study because mini-invasive harvesting is neither open nor endoscopic. Shapira et al. (2006a) divided all 54 patients into three groups: endoscopic, conventional, and conventional with harmonic shears harvesting [32]. Conventional harvesting and conventional harmonic shears harvesting were recognized as similar operating methods in these days, so they were combined as the OAH group in our study.

**Fig 1:**
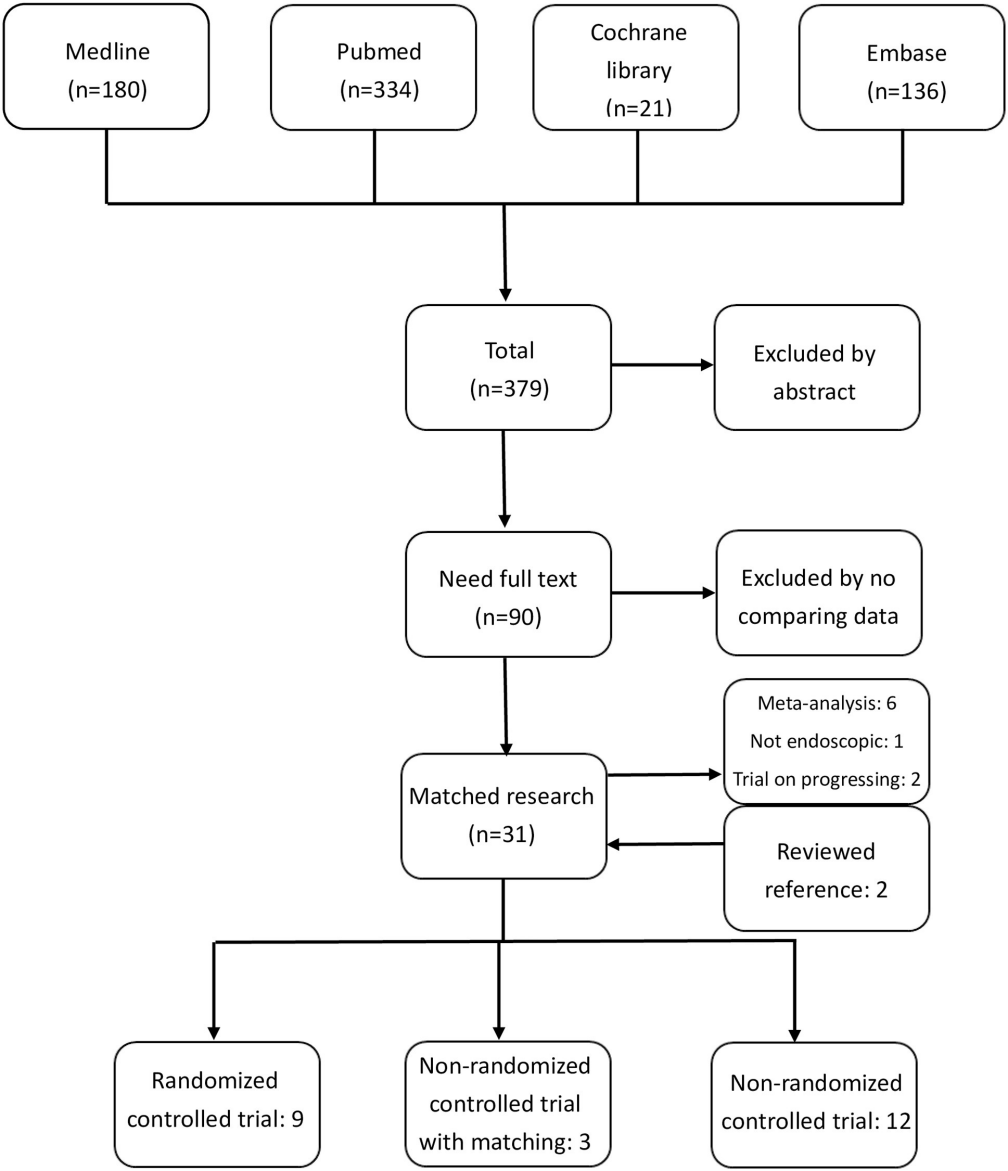
Study searching flow diagram.

The whole searching flow diagram is shown in Fig 1. In total, 4033 patients were included in 24 studies, while 1714 (42.5%) patients underwent EAH and 2319 (57.5%) underwent OAH. All patients included were modified Allen test-negative and had no obvious stenosis, dissection, or obstruction of the radial and ulnar artery upon duplex examination. The characteristics of the included studies are analyzed and shown in Tables 1 and 2. Details of radial artery harvesting, such as the harvesting time, length, wound incision, device, and air infusion assisting were recorded and are presented in Table 3. Almost all radial arteries were harvested with the pedicle method in these studies. However, it was not mentioned in some (4/18, 22.2%) of the studies [24, 36–39, 41–44, 47]. We performed a subgroup analysis of the three subgroups: RCT, NRCT with matching, and NRCT. We reported results of included studies in S3 Table.

**Table 1:** Randomized control trail comparing endoscopic and conventional radial artery harvesting.

Author (Year) [Reference]	Study Period	Country	Type of Study	Inclusion Criteria	Outcome	Note	f/u: month(SD)
Shapira et al. (2006a) [32]	<2005	United States	RCT	First-time isolated CABG	Histological changes, mortality, post-op MI, stroke, wound complication	Three groups (harmonic shears), pathology, and velocity	Until discharge
Rudez et al. (2007) [31]	2002/10~2004/10	Croatia	RCT	CABG patients	Mortality, neurological complication, wound pain, ulnar artery flow	Post operation echo for ulnar artery	37(7)
Grus et al. (2011) [30]	2005/01~2007/12	Czech Republic	RCT	Exclusion criteria by doppler: occluded RA, hypoplastic RA, chronic dissection of RA, stenotic subclavian artery, stenotic ulnar artery, patient’s on chronic dialysis	Wound, neurological complication, in-hospital mortality	Three groups (mini-invasive), RA used as Y-graft together with LIMA	Until discharge (about 10 days)
Nowicki et al. (2011) [29]	2004/1/1~ 2007/12/31	Poland	RCT	Exclusion criteria: 1. age > 70; 2. body weight >95 kg; 3. need of bilateral artery or additional saphenous vein; 4. abnormal Allen test; 5. coexistence of chronic renal failure or renal disease	Graft occlusion, endothelium with CD31, eNOS stain	Outcome measured by immunohistochemical analysis. CD31, eNOS staining patency measured by sonography	36
Kiaii et al. (2017) [25] Burns et al. (2015) [26] Burns et al. (2014) [27] Kiaii et al. (2013) [28]	2005/04~2007/01	Canada	RCT	1. coronary artery disease, need elective or urgent CABG; 2. age > 18; 3. Allen test negative	Pain, hospital days, wound, neurological complication, mortality, patency, quality of life	Urgent operation, EAH vs. OAH: 48.3% vs. 54.2%	60
Tamim et al. (2017) [24]	2013~2016	Saudi Arabia	RCT	Nonemergent on-pump CABG	Patency	f/u patency by 64-slice cardiac CT, only abstract	12
Navia et al. (2012) [34] Navia et al. (2011) [35]	2002/01~2004/07	United States	NRCT, matching.	Inclusion criteria: patient receiving CABG Exclusion criteria: 1. Allen test positive or perfusion index <45%; 2. Raynaud disease, Dupuytren contracture, rheumatoid arthritis, subclavian stenosis, and renal failure needing H/D	Wound complication, neurological complication, mortality, organ failure	N/A	Until discharge
Bisleri et al. (2016) [33]	2005/01~2014/01	Italy	NRCT, matching.	Patient receiving on-pump total arterial CABG surgery. At least 85%~90% target stenosis	Mortality, neurological complication, MACE, patency	N/A	60
Galajda et al. (2002) [47]	1999/01~2000/12	Hungary	Retrospective study	CABG patients	Mortality, wound, neurological complication	Both side RA of 200 patients	12
Patel et al. (2004) [46]	Before 2004	United States	Prospective, NRCT	Primary CABG Exclusion criteria: incomplete palmar arch, no compensatory flow, or renal failure	Neurological complications, wound complications	All case harvesting unilateral side RA	6
Shapira et al. (2006b) [45]	2002/12~2004/06	United States	Retrospective study	CABG patients	Mortality, MACE, wound complication	N/A	1
Bleiziffer et al. (2007) [44] Bleiziffer et al. (2008) [42]	2004/03~2005/07	Germany	Retrospective study	CABG patients Exclusion criteria: 1. age > 70; 2. abnormal Allen test or duplex finding; 3. coexistence of chronic renal disease, carpal tunnel syndrome, M. Dupuytren, severe arterial obstructive disease, visible calcification of RA	Neurological complications, long-term patency rate.	In 2007 study, three patients were excluded due to contraindication of application of contrast agent OAH group was randomly and retrospectively selected f/u by CT	12
Burris et al. (2008) [41]	2004/06~2007/05	United States	Retrospective study	Isolated off-pump CABG Exclusion criteria: creatinine level exceeding 2.0 mg/dL, abnormal Allen test, under hemodialysis, uncontrolled diabetes mellitus or Raynaud disease	Pathological patency	Checked by optical coherence tomography CT angiogram for patency, EAH, OAH group had cadaver artery (20, 4)	Until discharge
Kim et al. (2007) [43]	2000/04~2005/07	Korea	Retrospective study	Primary CABG, RA as secondary conduit Exclusion criteria: 11 died within 6 months and 4 lost f/u	Wound, neurological complication, short-term patency	OAH during 2000/04~2003/04. EAH during 2003/05~2005/07 Patency rate by MDCT	6
Medalion et al. (2008) [40]	before 2008	Israel	Prospective, NRCT	First-time isolated CABG	MACE, wound infection, RA vasoreactivity and relaxation.	Vasoreactivity and relaxation checked by histological examination, H&E, Masson trichrome and Verhoeff van Gieson’s stains	Until discharge
Ito et al. (2009) [39]	2006/02~2008/03(EAH) 2003/08~2005/11(OAH)	Japan	Prospective, NRCT	Patient receiving CABG surgery, age <75 Exclusion criteria: chronic kidney disease, Allen test positive, RA diameter <2 mm	Wound, neurological complication, mortality, MACE, ICU stay, hospital stay	EAH was done by prospective study and retrospective data for control group (OAH), patency measured by angiography, patency rate counted by anastomosis	Until discharge
Ito et al. (2011a) [36] Ito et al. (2011b) [37]	1999/04~2009/12	Japan	Retrospective study	Isolated off-pump CABG	Mortality, cardiac death, cardiac event, short-term and long-term patency rate	N/A	36
Dimitrova et al. (2010) [38]	2000/02~2008/01(EAH) 1995/01~2000/01(OAH)	United States	Retrospective study	Patient receiving CABG and receiving postoperative angiography	Mortality, MACE, long-term patency rate	Post-operation angiography Total patient with EAH/OAH: 727/724, included in patency rate study: total 202	EAH/OAH: 36(24)/78.3(40)

CABG, coronary artery bypass grafting; CT, computed tomography; EAH, endoscopic radial artery harvesting; f/u, follow-up time; H/D, hemodialysis; ICU, intensive care unit; LIMA, left internal mammary artery; MACE, major adverse cardiac events; MDCT, multidetector computed tomography; MI, myocardial infarction; NRCT, non-randomized controlled trial; OAH, open radial artery harvesting; RA, Radial artery; RCT, randomized controlled trial; SD, standard deviation. Ps: Different methods were used for the outcome measurement in each study: (a) by pathology: Shapira et al. (2006a); (b) by computed tomography: Tamim et al. (2017), Bleiziffer et al. (2007), (2008), Burris et al. (2008), and Kim et al. (2007); (c) by angiography: Kiaii et al. (2017), Ito et al. (2009), and Dimitrova et al. (2010); (d) by sonography: Nowicki et al. (2011).

**Table 2:** The key characteristics of the included studies.

Author (Year) [Reference]	EAH/OAH	Patient Number	Age(SD)	Gender (male)	LVEF (%) (SD)	HTN	DM	CKD	Prior CVA	PAOD	Urgent Operation
Shapira et al. (2006a)	EAH	18	60(10)	88.9%	53(13)	72.2%	44.4%	0.0%	11.1%	22.2%	N/A
	OAH	18	58(12)	77.8%	51(14)	83.3%	61.1%	5.6%	11.1%	33.3%	N/A
Rudez et al. (2007)	EAH	25	60.5(9.2)	64.0%	N/A	56.0%	32.0%	N/A	N/A	N/A	N/A
	OAH	25	61.2(9.8)	72.0%	N/A	60.0%	24.0%	N/A	N/A	N/A	N/A
Grus et al. (2011)	EAH	20	55.7(6.8)	90.0%	N/A	60.0%	55.0%	5.0%	N/A	N/A	N/A
	OAH	20	53.3(4.6)	95.0%	N/A	65.0%	60.0%	10.0%	N/A	N/A	N/A
Nowicki et al. (2011)	EAH	100	55.7	88.0%	N/A	N/A	20.0%	0.0%	N/A	10.0%	N/A
	OAH	100	59.7	91.0%	N/A	N/A	18.0%	0.0%	N/A	12.0%	N/A
Kiaii et al. (2017) Burns et al. (2015) Burns et al. (2014) Kiaii et al. (2013)	EAH	60	57.8(6.8)	90.0%	CHF:1.7%	N/A	25.0%	N/A	5.0%	0.0%	48.3%
	OAH	59	57.9(7.9)	93.2%	CHF:1.7%	N/A	20.3%	N/A	0.0%	2.0%	54.2%
Tamim et al. (2017)	EAH	15	N/A	N/A	N/A	N/A	N/A	N/A	N/A	N/A	N/A
	OAH	15	N/A	N/A	N/A	N/A	N/A	N/A	N/A	N/A	N/A
Navia et al. (2012) Navia et al. (2011)	EAH	39	60(9.9)	38.9%	50(13)	76.9%	17.9%	2.6%	N/A	41.0%	N/A
	OAH	117	62(9.1)	94.9%	47(13)	78.6%	18.8%	1.7%	N/A	42.7%	N/A
Bisleri et al. (2016)	EAH	82	65.1(11.3)	79.2%	EF<40%: 15.8%	73.1%	23.1%	8.5%	N/A	26.8%	N/A
	OAH	82	66.5(7.3)	71.9%	EF<40%: 19.8%	67.9%	34.6%	3.7%	N/A	34.6%	N/A
Galajda et al. (2002)	EAH	50	N/A	N/A	N/A	N/A	N/A	N/A	N/A	N/A	N/A
	OAH	465	N/A	N/A	N/A	N/A	N/A	N/A	N/A	N/A	N/A
Patel et al. (2004)	EAH	100	69	71.0%	N/A	76.0%	39.0%	N/A	N/A	N/A	N/A
	OAH	100	68	66.0%	N/A	71.0%	31.0%	N/A	N/A	N/A	N/A
Shapira et al. (2006b)	EAH	108	61(9)	87.0%	50(15)	87.0%	42.6%	N/A	5.6%	15.7%	63.9%
	OAH	120	62(9)	76.7%	50(14)	77.5%	37.5%	N/A	10.0%	18.3%	76.7%
Bleiziffer et al. (2007) Bleiziffer et al. (2008)	EAH	50	60.1(6.7)	88.0%	56.5(7.8)	80.0%	12.0%	0.0%	N/A	8.0%	N/A
	OAH	50	59.2(8.2)	84.0%	54.0(10.8)	83.0%	20.0%	0.0%	N/A	12.0%	N/A
Burris et al. (2008)	EAH	21	66.0(11.6)	66.7%	N/A	71.4%	38.1%	0.0%	4.8%	14.3%	N/A
	OAH	39	64.4(10.6)	71.8%	N/A	89.7%	38.5%	0.0%	5.1%	15.4%	N/A
Kim et al. (2007)	EAH	100	63.3(8.2)	77.0%	48.5(10.8)	48.0%	48.0%	N/A	N/A	N/A	N/A
	OAH	157	59.8(8.8)	65.6%	50.5(9.2)	45.9%	39.5%	N/A	N/A	N/A	N/A
Medalion et al. (2008)	EAH	40	62(10)	87.5%	LVF: 5%	80.0%	37.5%	N/A	N/A	N/A	N/A
	OAH	40	66(10)	82.5%	LVF: 5%	80.0%	30.0%	N/A	N/A	N/A	N/A
Ito et al. (2009)	EAH	50	62.8(9.1)	84.0%	59.5(12.9)	66.0%	52.0%	0.0%	8.0%	N/A	N/A
	OAH	50	62.8(9.4)	82.0%	62.8(11.6)	78.0%	56.0%	0.0%	16.0%	N/A	N/A
Ito et al. (2011a) Ito et al. (2011b)	EAH	109	N/A	N/A	N/A	N/A	N/A	N/A	N/A	N/A	N/A
	OAH	138	N/A	N/A	N/A	N/A	N/A	N/A	N/A	N/A	N/A
Dimitrova et al. (2010)	EAH	727	57.6	84.0%	EF<40%: 22%	64.0%	37.0%	1.3%	6.0%	6.0%	77.0%
	OAH	724		82.0%	EF<40%: 21%	64.0%	36.0%	1.2%	4.0%	5.0%	77.0%

CHF, congestive heart failure; CKD, chronic kidney disease or end stage renal disease; CVA, cerebral vascular disease; DM, diabetes mellitus; EAH, endoscopic radial artery harvesting; EF, ejection fraction; HTN, hypertension; LVF, left ventricle failure; N/A, not available; OAH, open radial artery harvesting; PAOD, peripheral artery occlusion disease; SD, standard deviation.

Ps1: Grus et al. (2011) excluded all patients under chronic dialysis. Chronic kidney disease was not excluded.

Ps2: Shapira et al. (2006b) recorded urgent and emergent operations, and we summarized the data as non-elective operations.

Ps3: Kiaii et al., Bisleriet et al., Mesalion et al., and Dimitrova et al. had no report on the ejection fraction of their patient, and they presented the heart function by recording the percentage of congestive heart failure, left ventricle failure, or an ejection fraction of less than 40%.

**Table 3:** Operation details of included studies.

Author (Year) [Reference]	EAH/OAH	Time (mins)	Wound (cm)	Length of Artery (cm)	Device	Air Infusion Assisting
Shapira et al. (2006a)	EAH	61(24)	3	N/A	1. CardioVations. 2. Ultra-Cision Harmonic Scalpel	N/A
	OAH	41(10)	N/A	N/A	No-touch, method published by Reyes et al.	nil
Rudez et al. (2007)	EAH	N/A	5	N/A	1. CardioVations, Sommerville. 2. Ethicon Endo-surgery ultrasonic scissors	CO2, 5 L/min
	OAH	N/A	N/A	N/A	Ultracision harmonic scalpel	nil
Grus et al. (2011)	EAH	52.6(11.3)	3	17.2(1.5)	1. ClearGlide Endoscopiv Vessel Harvesting system, Datascope. 2. UltraCision Harmonic Scalpel	CO2, 8–10 L/min
	OAH	27.8(4.6)	N/A	18.5(0.7)	Harmonic Scalpel	nil
Nowicki et al. (2011)	EAH	72.7(23.4)	2	16.0(2.2)	Guidant VasoView 6	
	OAH	40.7(5.7)	N/A	15.1(2.2)	No-touch, method published by Reyes et al.	nil
Kiaii et al. (2017) Burns et al. (2015) Burns et al. (2014) Kiaii et al. (2013)	EAH	N/A	2	N/A	1. ESVH retractor (Karl Storz, Tuttlingen, Germany). 2. Harmonic shears (Ethicon EndoSurgery)	No CO2 assisting
	OAH	N/A	N/A	N/A	Metzenbaum scissors and electrocautery	nil
Tamim et al. (2017)	EAH	N/A	N/A	N/A	N/A	N/A
	OAH	N/A	N/A	N/A	N/A	nil
Navia et al. (2012) Navia et al. (2011)	EAH	33(13)	2~3	N/A	VASOVIEW Endoscopic Vessel Harvesting System (Maquet)	N/A
	OAH	N/A	N/A	N/A	Method published by Reyes et al.	nil
Bisleri et al. (2016)	EAH	41.7(18.3)	2.5	N/A	Endoscopic RA retractor (Kal Storz, Tuttlingen) Ligasure (covidien), Hook (Stoz)	N/A
	OAH	36.5(15.7)	20	N/A	Method published by Reyes et al.	nil
Galajda et al. (2002)	EAH	30	2	N/A	Harmonic scalpel	N/A
	OAH	30	N/A	N/A	S shape, clips for preventing thermic injury, Redon drain	nil
Patel et al. (2004)	EAH	26	2.8	18.1	VASOVIEW System (Guidant Corporation)	CO2, 15 mmHg
	OAH	22	24.6	17.2	N/A	nil
Shapira et al. (2006b)	EAH	61(24)	3	19.7(2.2)	Ultra-Retractor (CardioVations, Somerville), UltraCision Harmonic scalpel	CO2
	OAH	45(11)	N/A	20.1(1.7)	Method published by Reyes et al.	nil
Bleiziffer et al. (2007) Bleiziffer et al. (2008)	EAH	N/A	3	N/A	Retractor, harmonic scalpel	N/A
	OAH	N/A	N/A	N/A	N/A	nil
Burris et al. (2008)	EAH	N/A	N/A	N/A	Vasoview6; Guidant Systems	CO2, 10 mmHg
	OAH	N/A	N/A	N/A	N/A	nil
Kim et al. (2007)	EAH	N/A	2~3	N/A	Vasoview; Guidant Systems	CO2, 10~15 mmHg
	OAH	N/A	N/A	N/A	Method published by Reyes et al.	nil
Medalion et al. (2008)	EAH	N/A	2~3	N/A	Method published by Connolly et al.	N/A
	OAH	N/A	N/A	N/A	Method published by Reyes et al.	nil
Ito et al. (2009)	EAH	27.4(6.5)	2.5	16.8(1.3)	VasoView System (version 4, Boston Scientific) Tourniquet inflated to 250 mmHg	CO2, 12 cm H2O
	OAH	N/A	N/A	18.5(1.5)	N/A	nil
Ito et al. (2011a) Ito et al. (2011b)	EAH	30.6	N/A	18.2	N/A	N/A
	OAH		N/A	16.2	N/A	nil
Dimitrova et al. (2010)	EAH	N/A	2	N/A	Sorin endovein harvest system Endoloop ligature	CO2
	OAH	N/A	15~25	N/A	No-touch	nil

EAH, endoscopic radial artery harvesting; N/A, not available; OAH, open radial artery harvesting. Ps: Almost all studies harvested the radial artery as a pedicle. Galajda et al. (2002), Bleiziffer et al. (2007), (2008), Ito et al. (2009), (2011a), (2011b), and Dimitrova et al. (2010) did not record the details of radial artery grafts.

#### Quality *a*ssessment

**Fig 2:**
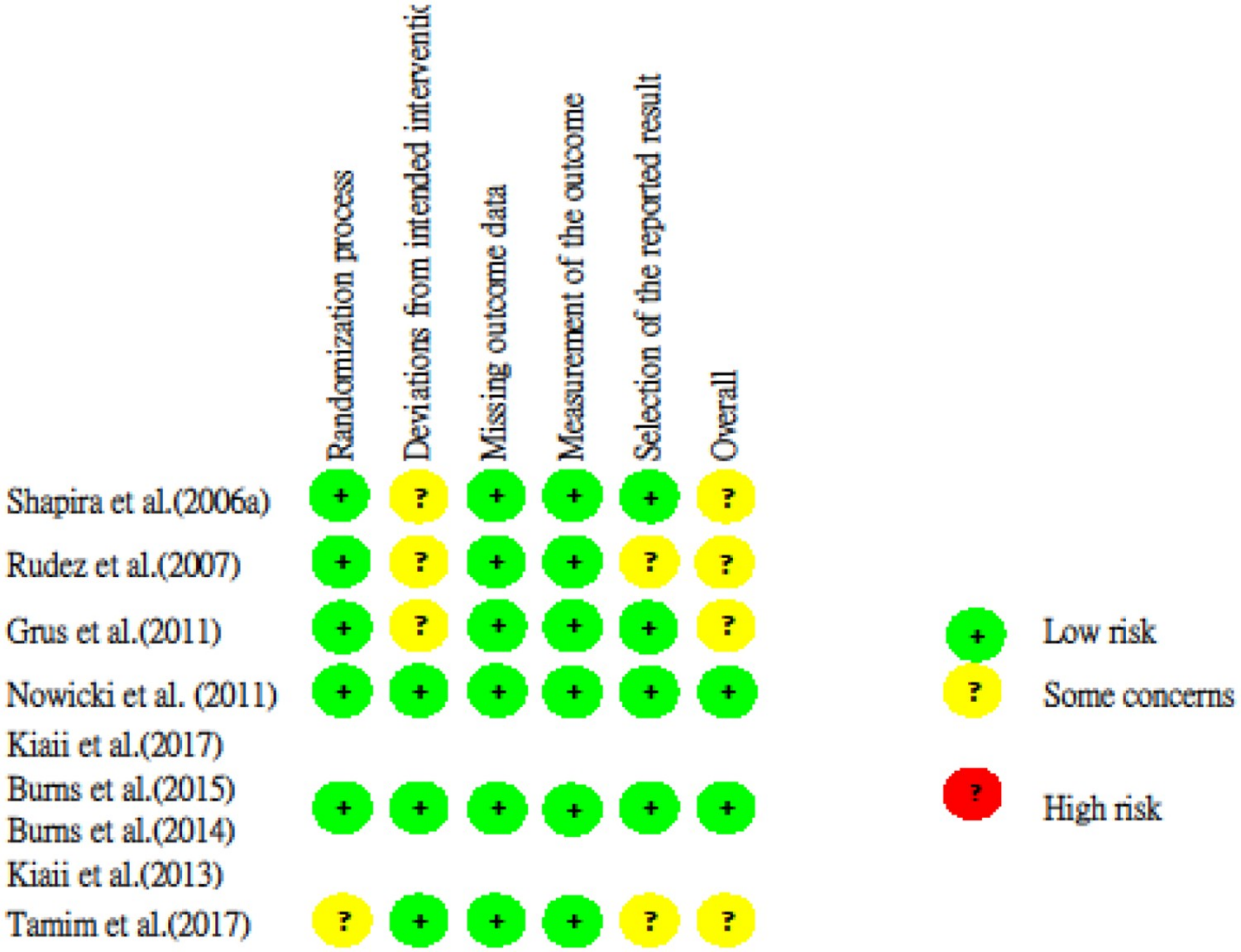
Risk of bias analysis for included Randomized Controlled Trials (RCTs).

The risk of RCTs was evaluated by RoB2, shown in Fig 2. Most of them were “low risk” or “some concerns”. The risk of bias of all NRCTs is shown in Table 4. The NOS of all trials ranged from 5 to 9 points. Ten NRCTs are high-quality studies (NOS ≧ 7 points).

**Table 4:** The Newcastle–Ottawa Scale of included non-randomized control studies.

Author (Year) [Reference]	Selection	Comparability	Outcome	Overall
Navia et al. (2012) Navia et al. (2011)	4	2	2	8
Bisleri et al. (2016)	4	2	3	9
Galajda et al. (2002)	3	1	3	7
Patel et al. (2004)	3	0	2	5
Shapira et al. (2006b)	4	0	3	7
Bleiziffer et al. (2007) Bleiziffer et al. (2008)	4	2	3	9
Burris et al. (2008)	4	2	2	8
Kim et al. (2007)	4	0	3	7
Medalion et al. (2008)	4	1	2	7
Ito et al. (2009)	3	0	2	5
Ito et al. (2011a) Ito et al. (2011b)	3	1	1	5
Dimitrova et al. (2010)	4	0	2	6

### Wound over the harvesting site

#### Wound infection rate over the harvesting site

**Fig 3:**
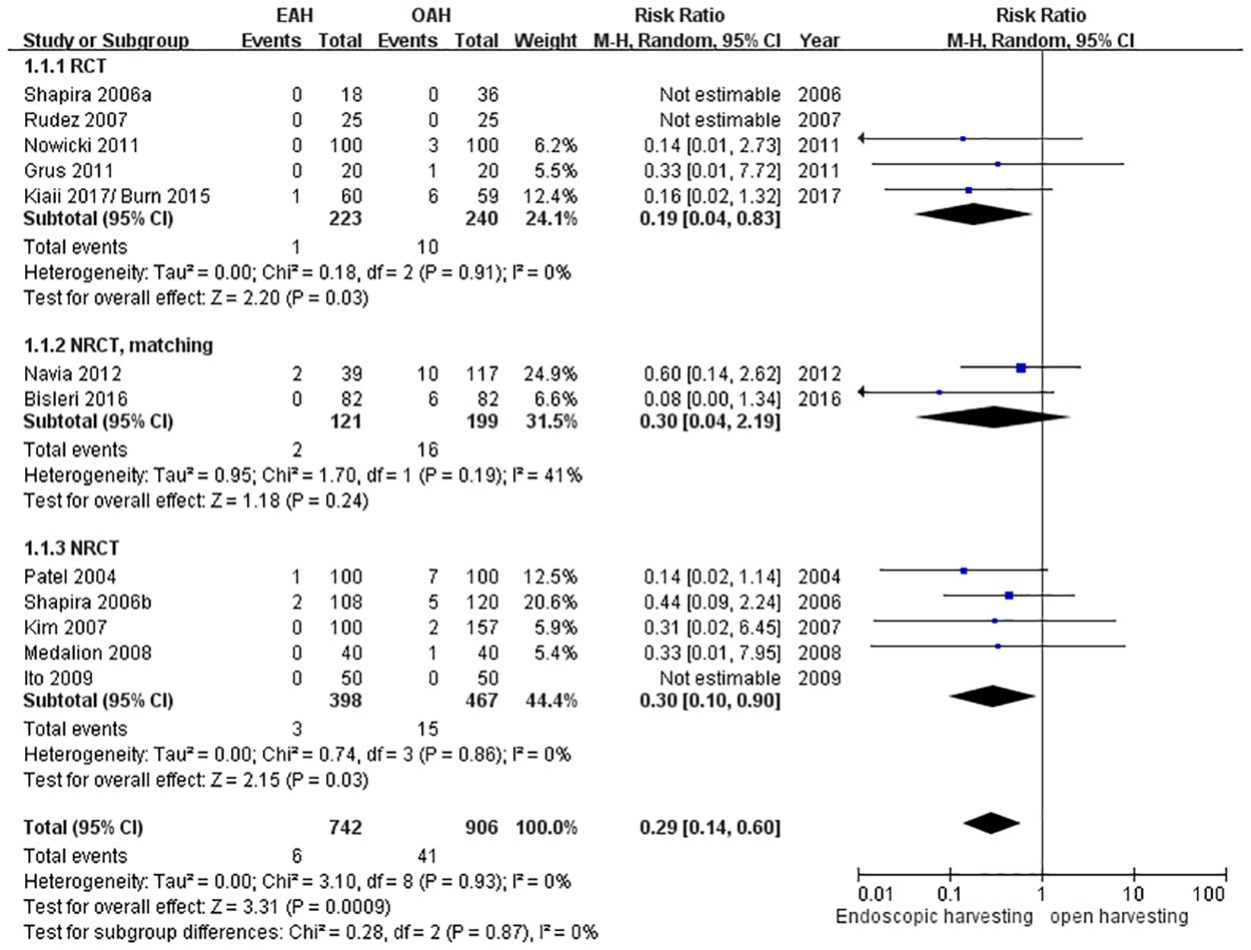
Forest plot for the wound infection rate.

Twelve studies were pooled into the analysis. The wound infection rate was significantly reduced by 71% (RR = 0.29, 95% CI = 0.14 to 0.60, *p* = 0.0009) (Fig 3). This benefit was demonstrated statistically for the NRCT (RR = 0.30, 95% CI = 0.10 to 0.90, *p* = 0.03) and RCT (RR = 0.19, 95% CI = 0.04 to 0.83, *p* = 0.03), but not for matched NRCT (RR = 0.30, 95% CI = 0.04 to 2.19, *p* = 0.24).

#### Wound complication rate over the harvesting site

**Fig 4:**
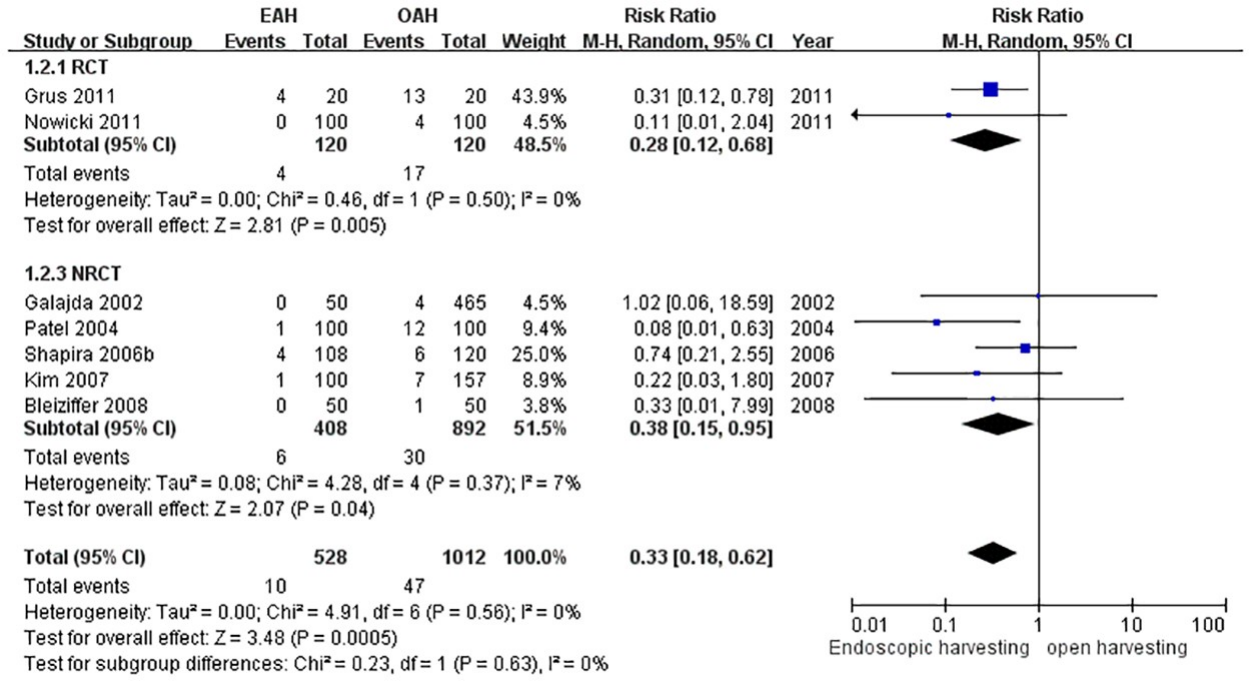
Forest plot for wound complications.

Wound complications were defined as more complicated wound conditions while healing, including hematoma, seroma, edema, poor wound edges healing, and wound infection. Complications were recorded in seven studies and were found to be significantly reduced in the EAH group (RR = 0.33, 95% CI = 0.18 to 0.62, *p* = 0.0005) (Fig 4).

### Neurological complication over the harvest site

**Fig 5:**
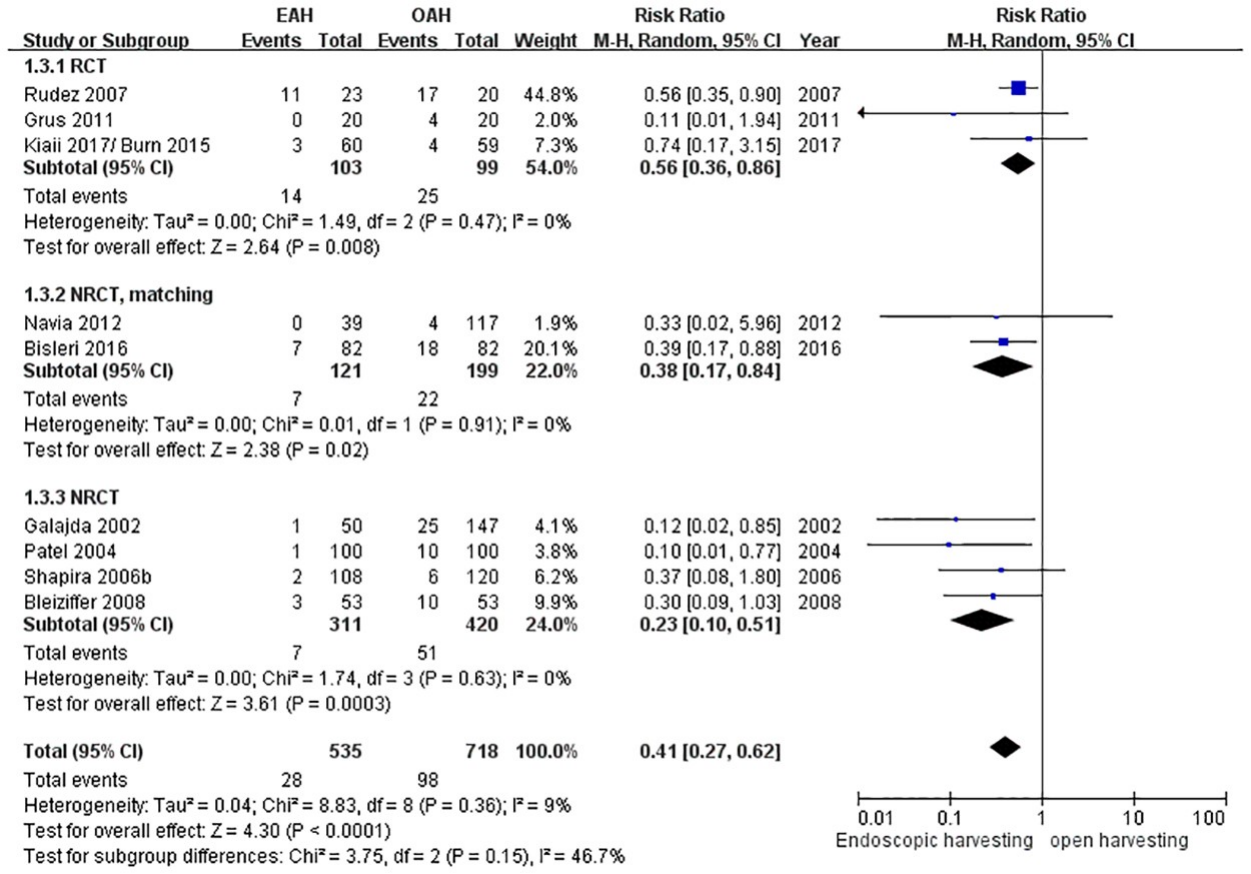
Forest plot for neurological complications.

Neurological complication over the harvesting site was defined as the impairment of sensitivity and mobility of the harvest sites, which was discussed in nine studies. The EAH group exhibited significantly decreased neurological complications over the harvest site, with a reduction of 59% (RR = 0.41, 95% CI = 0.27 to 0.62, *p* < 0.0001; Fig 5).

### In-hospital mortality or 30-day mortality

**Fig 6:**
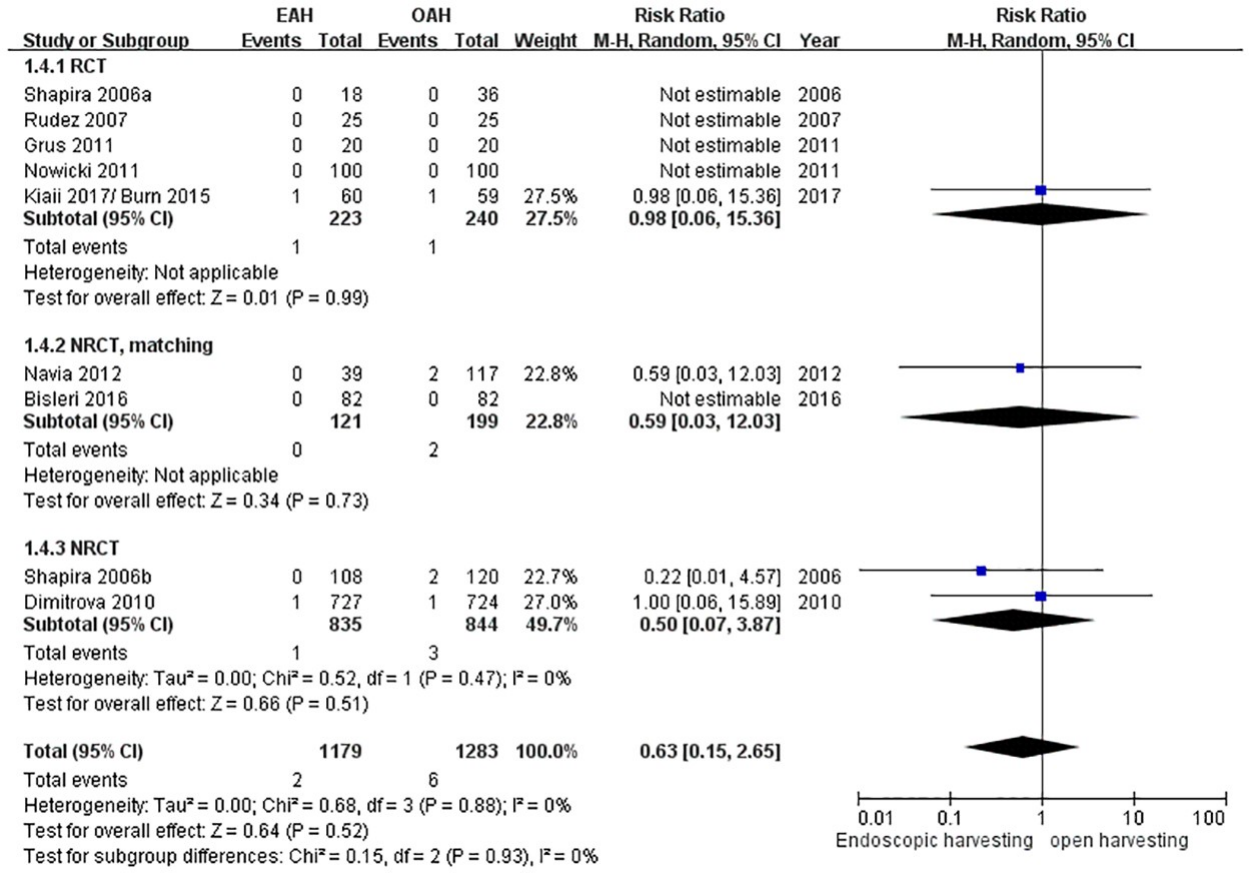
Forest plot for in-hospital mortality or 30-day mortality.

Nine included studies made in-hospital mortality or 30-day mortality a secondary outcome. However, the mortality events occurred in only four studies. There is no significant difference between the two groups (RR = 0.63, 95% CI = 0.15 to 2.65, *p* = 0.52) (Fig 6). These studies have low heterogeneity (I^2^ = 0%).

### Long-term survival (over one year)

**Fig 7:**
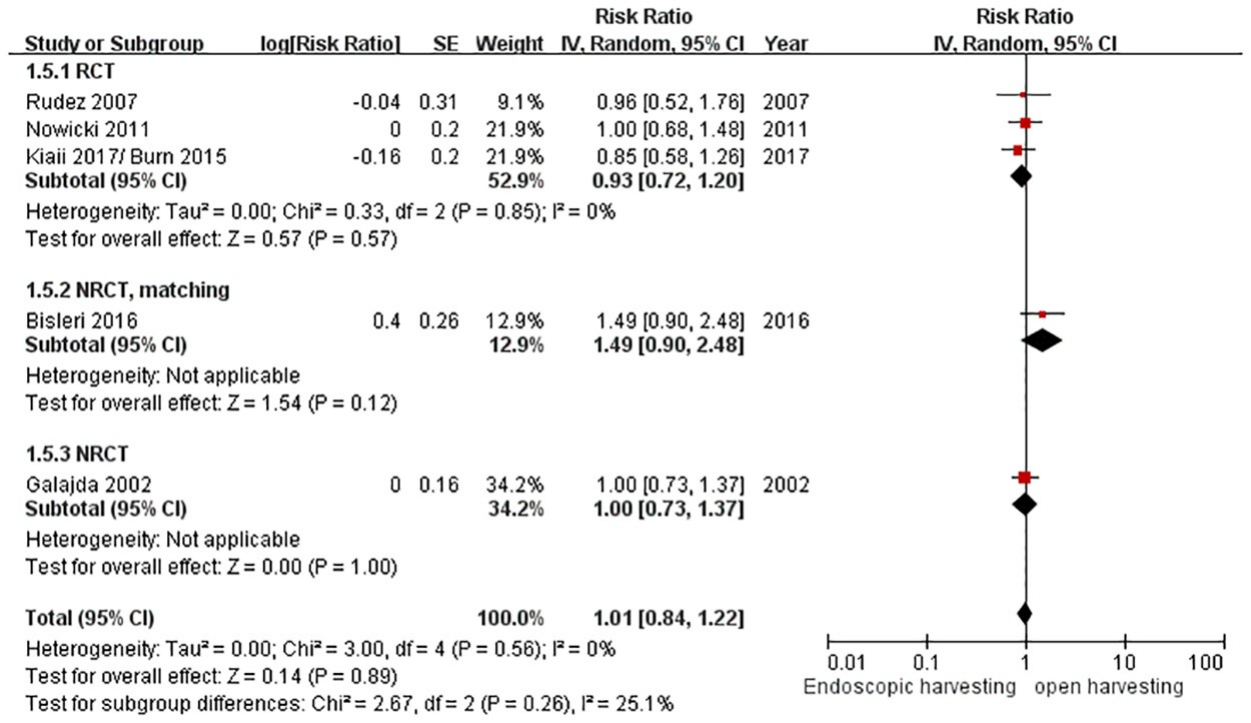
Forest plot for the long-term survival rate.

Only five studies provided long-term follow-up data for over one-year survival. Galajda et al. (2002) showed one-year survival [47]. Rodez et al. (2007) and Nowicki et al. (2011) followed survival over 3 years [29, 31]. Kiaii et al. (2017)/Burn et al. (2015) and Bisleri et al. (2016) reported the survival rate over 5 years [25, 26, 33]. There was no significant difference in long-term survival between these two groups (RR = 0.95, 95% CI = 0.78 to 1.16, *p* = 0.63) (Fig 7).

### Patency rate

We summarized four ways to evaluate the post-operation conduit patency rate from ten studies. In Tamim et al. (2017), Bleiziffer et al. (2007), (2008), Burris et al. (2008), and Kim et al. (2007), the patency rates were measured by computed tomography (CT) [24, 41–44]. In Kiaii et al. (2017)/Burns et al. (2015), Ito et al. (2009), (2011), and Dimitrova et al. (2010), the patency rates were measured by angiography [25, 26, 36–39]. Nowicki et al. (2011) evaluated their patency rate by sonography follow-up [29]. Shapira et al. (2006a) measured the conduit patency rate by pathology studies, but the pathology report was only for the harvested conduit itself [32]. Therefore, we excluded Shapira et al. (2006a) and compiled a total of nine studies for the statistics of patency rates.

All these studies had different follow-up times. Kim et al. (2007) did not define the timing of following up and only mentioned that it was shortly after CABG [43]. Ito et al. (2009) only followed angiography before patients were discharged from the hospital [39]. Burris et al. (2008) followed CT one week after CABG operation [41]. Tamim et al. (2017) followed CT at 6 and 12 months after the operation [24]. Bleiziffer et al. (2007), (2008) checked CT at 12 months after the operation [42, 44]. Ito et al. (2011), Dimitrova et al. (2010), and Nowicki et al. (2011) checked the patency rate at 3 years after the operation [29, 36–38]. Kiaii et al. (2017)/Burns et al. (2015) followed patients for over 5 years [25, 26].

**Fig 8:**
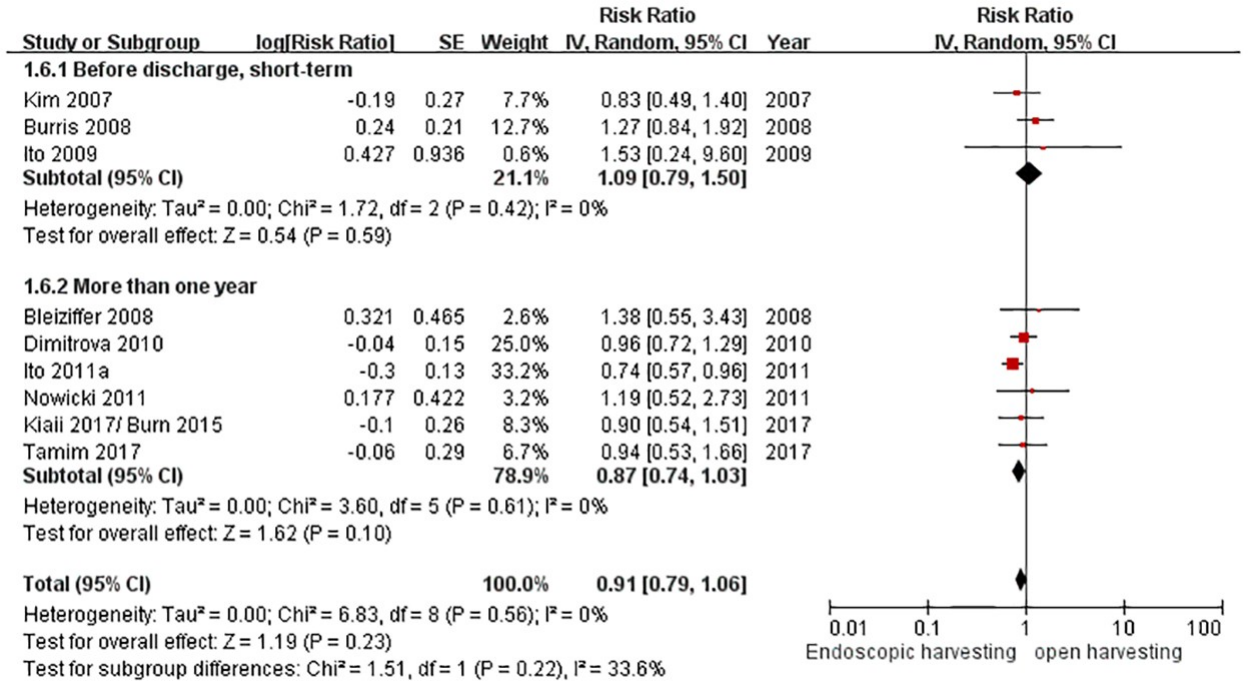
Forest plot for the patency rate.

We divided these nine studies, included in our patency rate study, into two groups by the follow-up period: “more than (or equal to) one year follow-up” and “before discharge, or unspecific short-term follow-up”. There was medium heterogeneity and no significant difference in the two groups (RR = 0.91, 95% CI = 0.79 to 1.06, *p* = 0.23, I^2^ = 33.6%) (Fig 8). However, the group that followed up for more than (or equal to) one year was “nearly” significant and indicated the advantage of EAH (RR = 0.87, 95% CI = 0.74 to 1.03, *p* = 0.10).

## Discussion

The earliest meta-analyses on the same subject were in 2014 and both came to the same conclusion that ERH had lower wound infection rate as well as less complications over harvesting site [48, 49]. As ERH was still a new technique back in 2014, there had been few long-term follow-up results that could help researchers investigate long-term survival. However, long-term outcomes are of great clinical interest for cardiac surgeons when assessing surgical approach of CABG. Therefore, although similar comparisons have been made by various researchers since 2014, we still believe that as more experiences are shared and higher volumes of ERH are accumulated, further research has its merits especially in observing long-term survival, which has become one of our key dimensions in the review process.

**Fig 9:**
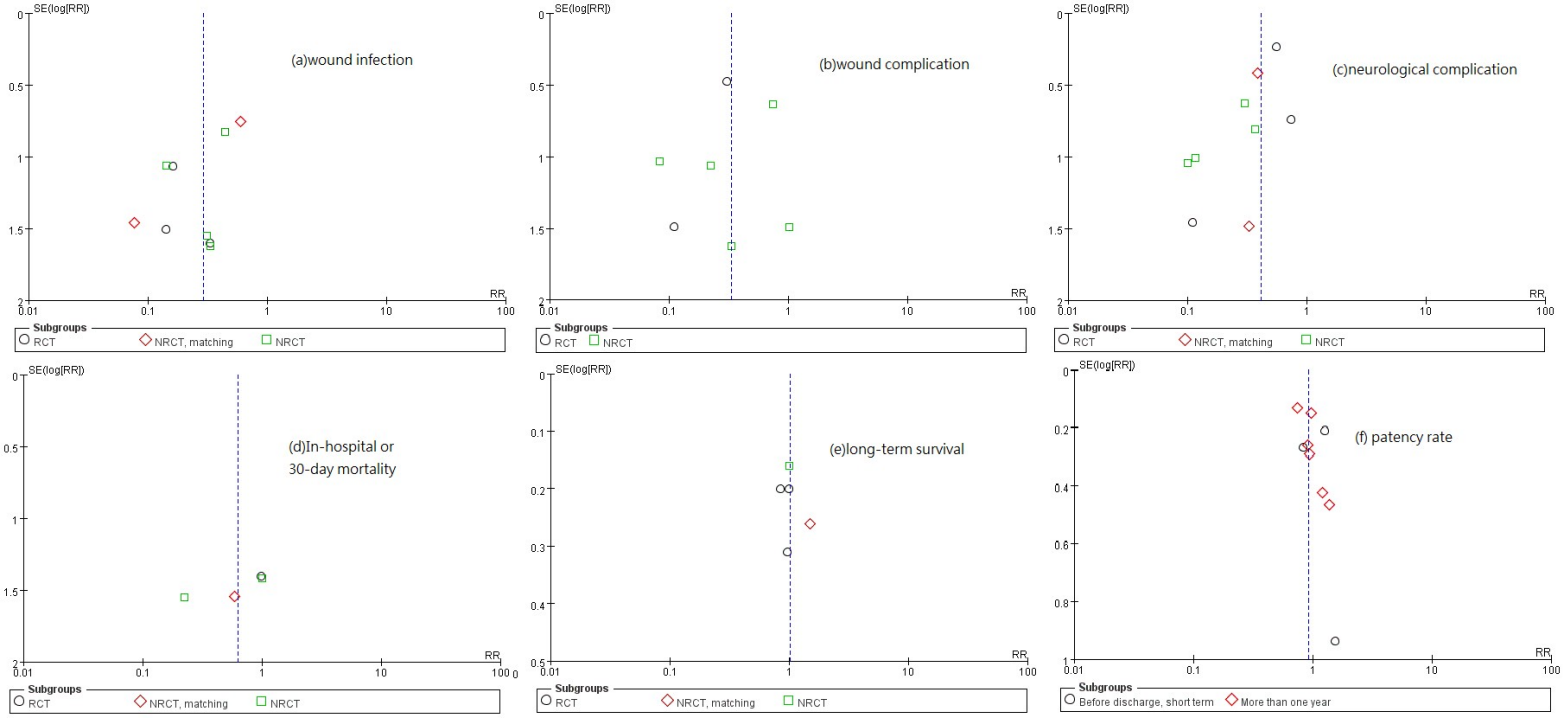
Funnel plot for publication bias: (a) wound infection, (b) wound complications, (c) neurological complications, (d) in-hospital or 30-day mortality, (e) long-term survival, and (f) patency rate.

Our analysis agreed to the results of Cao et al. in wound infection over harvesting site, wound complications over harvesting site, neurological complications over harvesting site, in-hospital or 30-day mortality, and patency rate (so did Wu et al. but they did not investigate neurological complications over harvesting site.) Yet Cao et al. have remarked that their studies were not randomized and their follow-up periods were rather short [49]. In order to highlight possible bias in patient selection, our analysis specifically separated RCT subgroups and non-RCT subgroups. Meanwhile, due to over 700 more patients included, our p-values are much lower so our findings indicated more significant differences between ERH and OAH in respect of wound infection over harvesting site, wound complications over harvesting site, and neurological complications over harvesting site. On occasions of lacking *p*-values or hazard ratios for clinical outcomes, we calculated necessary statistics based on the data provided in the literature and derived the ratios and values for further analysis. There were five meta-analysis studies published [7, 48–51]. All the articles included in them were also included in our study. The large number of patients included (total 4033) enhanced the credibility of our studies. We further reviewed them in six key dimensions of surgical outcomes in respect of both recovery and survival, and listed the comparisons with other studies in S4 Table. All publication biases of each study were analyzed and we generated a funnel plot to present the biases (Fig 9).

The use of multiple arterial conduits, including RA conduits, improved the long-term survival of patients who received CABG, which resulted from the finding of the long-term patency rate of arterial conduits being higher than that of venous grafts [6]. Previous studies showed that RA would be more beneficial in CABG patients with severe proximal coronary artery stenosis, especially where stenosis was more than 90% [16, 52, 53]. Dimitrova et al. reported the benefit of using RA grafts on target vessels with stenosis over 80%. OAH with a non-touch technique can prevent artery injuries or spasms [8, 14]. Recently, the minimal invasive incisions of EAH have encouraged more and more surgeons to adopt EAH. However, the possibility of “touching” RA during EAH has raised doubts that possible injuries on vessels might impact the patency rate and survival rate. The endoscopic procedure of EAH takes place in a very narrow space, raising the concern of potential mechanical injuries to the conduits, especially on the endothelium. A pathologic examination also showed that RA from OAH had a significantly higher vasodilation response to medications [54]. However, our statistical analysis suggested that there was no difference in the patency rate between OAH and EAH (RR = 0.91, 95% CI = 0.79 to 1.06, *p* = 0.23).

Another major concern about RA harvesting is forearm ischemia, which is a severe complication that may cause hand claudication or ischemic necrosis. Pre-operation examinations—the Allen test and duplex exam for the radial and ulnar artery—are very important. Complete pre-operation RA surveys were performed in these 24 studies and forearm ischemia was not noted in either the EAH or OAH group. Rudez et al. arranged a post-operation echo for ulnar artery assessment, 37 ± 7 months after the operation [31]. The flow (13.1 cm/s in the EAH group, 15.9 cm/s in the OAH group) and diameters of ulnar arteries increased. The compensatory flow of forearm arteries was well-developed. Additionally, there was no hand or forearm claudication in all patients included in our study.

Three other meta-analysis studies have reported EAH to be beneficial for decreasing wound infections [7, 50, 51]. Our study included more literature and demonstrated the significant benefit for preventing wound infections (RR = 0.29, 95% CI = 0.14 to 0.60, *p* = 0.0009). Furthermore, we also analyzed wound complications, such as hematoma, seroma, edema, or poor wound edges healing. (Two of previous studies had discussed wound complications but only mentioned wound hematoma [48, 49].) Our results support that EAH could decrease wound complications (RR = 0.33, 95% CI = 0.18 to 0.62, *p* = 0.0005). Although most of the wound complications would recover without surgical intervention, they would lead to a worse life quality and worse post-operation care compliance [30].

There are nerves anatomically located at the RA harvesting site. Nerve injury is one of the main concerns of RA dissection. The superficial radial nerve is closely related to RA at the distal forearm and the superficial portion of the lateral antebrachial cutaneous nerve, which might be injured by the forearm wound incision [42]. Neurological complications over the harvesting site are defined as a weakness of fingers, sensory disturbances, numbness, or neuralgia [31, 42, 46]. Our study showed that EAH could decrease neurological complications (RR = 0.41, 95% CI = 0.27 to 0.62, *p* < 0.0001). Most of them recovered within 6 months [31, 46]. Wounds and neurological complications over the harvesting site worsen the quality of life of patients and delay the initial heart-lung rehabilitation course. Both situations hinder the feasibility of bilateral forearm RA harvesting and limit the harvesting site to the non-dominant arm. Our finding on EAH causing less forearm wounds and less neurological complications encourages bilateral forearm RA harvesting, allowing more arterial conduits to be available.

Only eight out of 2462 patients died in hospital or within 30 days, of which two underwent EAH and six underwent OAH. The in-hospital or 30-day mortality rate is insignificantly low for both groups, which supported the findings in the meta-analysis of Cao et al.[49], Wu et al.[48] and Ferdinand et al.[51]. However, their studies lacked follow-ups of long-term survival, which is an important indicator to decide the surgical approach as the main purpose of CABG is to prevent the mortality caused by heart failure in the future. We had investigated the long-term survival (over one year) and found a matched NRCT study, reported by Bisleri et al., showed that the OAH group had a trend avoiding cardiac-related mortality after 5-year follow-up, with statistical significance (*p* = 0.448) [33]. Nevertheless, consolidating the data from all the studies we included, there is no significant difference between these two groups (RR = 1.01, 95% CI = 0.84 to 1.22, *p* = 0.89), which should offer a different view regarding the doubts of EAH being inferior.

The RA graft patency rate was evaluated by three different methods: computed tomography, angiography, and sonography. We found no significant difference between the EAH and OAH group (RR = 0.91, 95% CI = 0.79 to 1.06, *p* = 0.23). We then further re-arranged the subgroups by follow-up period: “more than (or equal to) one year follow-up” and “before discharge, or unspecific short-term follow-up”. The outcome of the “more than (or equal to) one year” group showed a nearly significant difference, which favored EAH (RR = 0.87, 95% CI = 0.74 to 1.03, *p* = 0.10); therefore, should the number of patients increase, EAH might have the advantage of improving the patency rate. Our finding implied that the RA patency rate may display no difference for OAH and EAH in short-term follow-up, but whether the long-term patency rate of EAH is superior to that of OAH needs further study. More data associated with long-term follow-up (at least one year) are required to evaluate the long-term patency rate.

Financial benefits were also reported by Shapira et al., Grus et al., and Kiaii et al. [25, 30, 32]. The cost of the endoscopic harvesting system reported in these studies was around US $438 to US $655. The extra hospital fees for patients with wound infections or other complications, which can be significantly reduced by EAH according to our results, would be much higher than the cost of EAH itself.

Our limitation is the variation in surgical experience and the volume in each medical centers. Surgical experience and the volume are important factors affecting the outcome. EAH has a learning curve, and it needs experience or even training model for technique maturing [55–57]. Yoshizaki et al. suggested the safe limit of the limb ischemia to be 90minutes. However, they addressed that the harvesting time for their first case was 97minutes. Adequate training would reduce the harvesting time therefore also decrease limb ischemic time[57]. Connolly et al. and Casselman et al. suggested to gain proficiency in endoscopic vein harvesting before attempting EAH.[55, 56]. We would recommend operators to evaluate such before applying our results to the case.

Another limitation of our study is the lack of long-term outcomes. Most of the studies reported their results upon discharge from the hospital or within one-month after patients had received CABG. Besides, the results of the patency of RA examined by three different methods could also be biased. To clarify the beneficial effect of EAH on the long-term patency rate of RA grafts, a large-scale prospective randomized trial comparison of EAH and OAH is needed.

## Conclusions

In conclusion, EAH is beneficial for decreasing harvesting site wound infections and complications, as well as harvesting site neurological complications. However, there is no significant difference in the in-hospital mortality, long-term survival, or patency rate between OAH and EAH. Our meta-analysis indicated that EAH can improve the outcome of the harvesting site without affecting the mortality, long-term survival, or graft patency. According to our findings, bilateral arm RA harvesting by EAH could be considered due to the low harvesting site wound and neurological complication rate.

## Supporting information

S1 Table Search strategy for Embase, Pubmed, Medline, and Cochran. (PDF)

S2 Table PRISMA 2009 checklist. (PDF)

S3 Table Clinical results of the included studies. (DOCX)

S4 Table Comparison among meta-analysis studies of endoscopic radial artery harvesting. (DOCX)
