# Supplementary material for: Radial artery harvesting in coronary artery bypass grafting surgery—Endoscopic or open method? A meta-analysis
Source: PLoS One. 2020 Jul 24;15(7):e0236499. doi: 10.1371/journal.pone.0236499 (PMC7380611; doi:10.1371/journal.pone.0236499)
Supplement: S1 Table — (PDF) [file pone.0236499.s001.pdf]

1 endoscopic
2 tunnel
3 coronary artery bypass surgery
4 CABG
5 radial artery
6 harvest*
7 (#1 or #2)
8 (#3 or #4)
9 (#7 and #8 and #5 and #6)

Lmited: article, article in press, conference review, review
Clinical article, clinical trial, cohort analysis, comparative comparative study, controlled clinical trial, controlled study, human, intermethod comparison, major clinical study, methodology, practice guideline, prospective study, retrospective study.

Embase	136
Pubmed	334
Medline	180
Cochrane	21
Total	379

Except

No relation with our study or comparing data	343
Meta-analysis	6
No endoscopic harvesting	1
Trial on progressing	2

Including 22

Randomized control trial	9
Non-randomized controlled trial:	
Without matching:	10
Matching:	3

Add 2 non-randomized controlled trail by reviewing reference.
