# Supplementary material for: Radial artery harvesting in coronary artery bypass grafting surgery—Endoscopic or open method? A meta-analysis
Source: PLoS One. 2020 Jul 24;15(7):e0236499. doi: 10.1371/journal.pone.0236499 (PMC7380611; doi:10.1371/journal.pone.0236499)
Supplement: S3 Table — (DOCX) [file pone.0236499.s003.docx]

| **S3 Table.** **Clinical results of the included studies.** | | | | | | | | |
| --- | --- | --- | --- | --- | --- | --- | --- | --- |
| **Author (Year)** | **EAH/OAH** | **Number of patients** | **Wound infection cases** | **Number of wound complications** | **Number of Neurological complications** | **In-hospital or 30-day mortality** | **Long term survival**  **(log[Risk Ratio])** | **Graft patency (log[Risk Ratio])** |
| Shapira et al. (2006a) | EAH | 18 | 0 | N/A | N/A | 0 | N/A | N/A |
|  | OAH | 18 | 0 | N/A | N/A | 0 |  |  |
| Rudez et al. (2007) | EAH | 25 | 0 | N/A | 11 | 0 | -0.04 | N/A |
|  | OAH | 25 | 0 | N/A | 17 | 0 |  |  |
| Grus et al. (2011) | EAH | 20 | 0 | 4 | 0 | 0 | N/A | N/A |
|  | OAH | 20 | 1 | 13 | 4 | 0 |  |  |
| Nowicki et al. (2011) | EAH | 100 | 0 | 0 | N/A | 0 | 0 | 0.177 |
|  | OAH | 100 | 3 | 4 | N/A | 0 |  |  |
| Kiaii et al. (2017)  Burns et al. (2015)  Burns et al. (2014)  Kiaii et al. (2013) | EAH | 60 | 1 | N/A | 3 | 1 | -0.16 | -0.1 |
|  | OAH | 59 | 6 | N/A | 4 | 1 |  |  |
| Tamim et al. (2017) | EAH | 15 | N/A | N/A | N/A | N/A | N/A | -0.06 |
|  | OAH | 15 | N/A | N/A | N/A | N/A |  |  |
| Navia et al. (2011) | EAH | 39 | 2 | N/A | 0 | 0 | N/A | N/A |
|  | OAH | 117 | 10 | N/A | 4 | 2 |  |  |
| Bisleri et al. (2016) | EAH | 82 | 0 | N/A | 7 | 0 | -0.706 | N/A |
|  | OAH | 82 | 6 | N/A | 18 | 0 |  |  |
| Galajda et al. (2002) | EAH | 50 | N/A | 0 | 1 | N/A | 0 | N/A |
|  | OAH | 465 | N/A | 4 | 25 | N/A |  |  |
| Patel et al. (2004) | EAH | 100 | 1 | 1 | 1 | N/A | N/A | N/A |
|  | OAH | 100 | 7 | 12 | 10 | N/A |  |  |
| Shapira et al. (2006b) | EAH | 108 | 2 | 4 | 2 | 0 | N/A | N/A |
|  | OAH | 120 | 5 | 6 | 6 | 2 |  |  |
| Bleiziffer et al. (2008) | EAH | 50 | N/A | 0 | 3 | N/A | N/A | 0.321 |
|  | OAH | 50 | N/A | 1 | 10 | N/A |  |  |
| Burris et al. (2008) | EAH | 21 | N/A | N/A | N/A | N/A | N/A | 0.24 |
|  | OAH | 39 | N/A | N/A | N/A | N/A |  |  |
| Kim et al. (2007) | EAH | 100 | 0 | 1 | N/A | N/A | N/A | -0.19 |
|  | OAH | 157 | 2 | 7 | N/A | N/A |  |  |
| Medalion et al. (2008) | EAH | 40 | 0 | N/A | N/A | N/A | N/A | N/A |
|  | OAH | 40 | 1 | N/A | N/A | N/A |  |  |
| Ito et al. (2009) | EAH | 50 | 0 | N/A | N/A | N/A | N/A | 0.427 |
|  | OAH | 50 | 0 | N/A | N/A | N/A |  |  |

N/A: not available; EAH: endoscopic radial artery harvesting; OAH: open radial artery harvesting.
