# Supplementary material for: Radial artery harvesting in coronary artery bypass grafting surgery—Endoscopic or open method? A meta-analysis
Source: PLoS One. 2020 Jul 24;15(7):e0236499. doi: 10.1371/journal.pone.0236499 (PMC7380611; doi:10.1371/journal.pone.0236499)
Supplement: S4 Table — (DOCX) [file pone.0236499.s004.docx]

|  | Cao et al. | Wu et al. | Ferdinand et al. | Rahouma et al. | Naik et al. | Huang et al. |
| --- | --- | --- | --- | --- | --- | --- |
| published year | 2014 | 2014 | 2017 | 2017 | 2018 | Current study |
| Studies included | 12 | 10 | 10 | 6 | 6 | 24 |
| Patients included | 3314 | 2782 | 1368 | 743 | 743 | 4033 |
| Wound infection over harvesting site | RR=0.36 95% CI:0.16-0.82 p=0.01 | RR=0.31 95% CI:0.13-0.74 p=0.008 | RR=0.28 95% CI:0.13-0.63 p=0.002 | N/A | N/A | RR=0.29 95% CI: 0.14-0.60 p=0.0009 |
| Wound complications over harvesting site | Hematoma: RR=0.45 95% CI:0.26-0.77 p=0.004 | Hematoma: RR=0.18 95% CI:0.05-0.67 p=0.01 | N/A | RR=0.33 95% CI:0.14-0.77 p=0.01 | RR=0.33 95% CI:0.14-0.77 p=0.01 | RR=0.33 95% CI:0.18-0.62 p=0.0005 |
| Neurological complications over harvesting site | RR=0.77 95% CI:0.61-0.99 p=0.04 | N/A | RR=0.28 95% CI:0.13-0.62 p=0.08 | N/A | N/A | RR=0.41 95% CI:0.27-0.62 p < 0.0001 |
| In-hospital or 30-day mortality | RR=0.67 95% CI:0.17-2.55 p=0.55 | RR=1.06 95% CI:0.26-4.38 p=0.94 | N/A | RR=0.00 95% CI:-0.02-0.01 p=0.74 | N/A | RR=0.63 95% CI:0.15-2.65 p=0.52 |
| Survival more than one-year | N/A | N/A | N/A | Long term mortality: RR=0.67 95% CI:0.11-4.17 p=0.66 | N/A | RR=0.95 95% CI:0.78-1.16 p=0.63 |
| Graft patency rate | RR=1.04 95% CI:0.97-1.12 p=0.24 | RR=0.81 95% CI:0.54-1.21 p=0.30 | N/A | RR=1.32 95% CI:0.76-2.27 p=0.32 | N/A | RR=0.91 95% CI:0.79-1.06 p =0.23 |

**S4 Table. Comparison among meta-analysis studies of endoscopic radial artery harvesting.**

CI: confidence level; N/A: not available; RR: relative risk.
